# Microtubule Dynamics and Neuronal Excitability: Advances on Cytoskeletal Components Implicated in Epileptic Phenomena

**DOI:** 10.1007/s10571-020-00963-7

**Published:** 2020-09-14

**Authors:** Giuditta Gambino, Valerio Rizzo, Giuseppe Giglia, Giuseppe Ferraro, Pierangelo Sardo

**Affiliations:** Department of Experimental Biomedicine, Neuroscience and Advanced Diagnostics (Bi.N.D.), Sezione Di Fisiologia Umana G. Pagano, University of Palermo, Corso Tukory 129, Palermo, Italy

**Keywords:** Hyperexcitability, Microtubules, Epilepsy, Neurodevelopment, Cannabinoids, Neuroprotection

## Abstract

Extensive researches have deepened knowledge on the role of synaptic components in epileptogenesis, but limited attention has been devoted to the potential implication of the cytoskeleton. The study of the development of epilepsy and hyperexcitability states involves molecular, synaptic, and structural alterations of neuronal bioelectric activity. In this paper we aim to explore the neurobiological targets involved in microtubule functioning and cytoskeletal transport, i.e. how dynamic scaffolding of microtubules can influence neuronal morphology and excitability, in order to suggest a potential role for microtubule dynamics in the processes turning a normal neuronal network in a hyperexcited one. Pathophysiological alterations of microtubule dynamics inducing neurodegeneration, network remodeling and relative impairment on synaptic transmission were overviewed. Recent researches were reported on the phosphorylation state of microtubule-associated proteins such as tau in neurodegenerative diseases and epileptic states, but also on the effect of microtubule-active agents influencing cytoskeleton destabilization in epilepsy models. The manipulation of microtubule polymerization was found effective in the modulation of hyperexcitability. In addition, it was considered the importance of microtubules and related neurotrophic factors during neural development since they are essential for the formation of a properly functional neuronal network. Otherwise, this can lead to cognitive deficits, hyperexcitability phenomena and neurodevelopmental disorders. Lastly, we evaluated the role of microtubule dynamics on neuronal efficiency considering their importance in the transport of mitochondria, cellular elements fulfilling energy requirements for neuronal activity, and a putative influence on cannabinoid-mediated neuroprotection. This review provides novel perspectives for the implication of microtubule dynamics in the development of epileptic phenomena.

## Introduction: Epilepsy and Possible Neurobiological Targets

Epilepsy is a common, chronic neurological disorder, characterized by recurring, unprovoked seizures that often requires long-term therapy with antiepileptic drugs (AEDs), unless it appears as drug-refractory (Schmidt and Löscher 2005; Tischfield et al. 2010). Epileptogenesis comprises the sequence of events leading to abnormal, hypersynchronous discharge of specific populations of cortical neurons (Bromfield et al. 2006). Epileptic seizures are characterized by high-frequency bursts of action potentials hypersynchronously generated in a neuronal population, whose modification of bioelectric activity turns a normal neuronal network into a hyperexcitable one (Bromfield et al. 2006). Although studies on animal models and human tissue have suggested that the development of epilepsy and hyperexcitability states involve molecular, cellular and structural alterations (Rakhade and Jensen 2009), a clear understanding of mechanisms underlying structural changes in epilepsy has not been achieved yet. Among the experimentally induced epilepsies exploited for the investigation of underpinning mechanisms, temporal lobe epilepsy (TLE) originates from the alteration of normal discharge in the hippocampus (Téllez-Zenteno and Hernández-Ronquillo 2012); thus, this structure constitutes a brain area of particular interest in the field (Carletti et al. 2015; Gambino et al. 2018; Navas-Olive et al. 2020). Indeed, recent evidence report the paradoxical increase in structural re-organization and functional connectivity of limbic circuitry in patients with TLE (Bonilha et al. 2012).

Among the plethora of epilepsy-related neurobiological targets, research has been oriented mostly towards synaptic components, neurotransmitter mechanisms and/or metabolic pathways (Bialer et al. 2001; Scharfman 2007; Roseti et al. 2013; Maffei et al. 2015; Carletti et al. 2016a, 2018). The regulation of synaptic transmission is considered a key feature of numerous pathophysiological processes leading to seizures (Lepeta et al. 2016). Indeed, mutated synaptic proteins could be implied in pathways essential for presynaptic function, neurotransmission and general bioelectric homeostasis; thus, the so-called “synaptopathy” includes several inherited forms of epilepsy (Finelli et al. 2019). However, investigating on alternative molecular mechanisms is important to deepen knowledge on both etiology and pathophysiology of epilepsy, as well as to develop novel and more effective therapeutic strategies. Surprisingly enough, much work has been done on the role of synaptic components in the pathogenesis of epilepsy, but relatively little attention has been given to the potential role of the cytoskeleton. Only a few studies have investigated so far the putative role of the cytoskeleton on epilepsy. Indeed, deficiencies in cytoskeleton-related dynamics underpin several neurodevelopmental alterations and neurodegenerative disorders (Millecamps and Julien 2013). The neuronal cytoskeleton physiologically regulates core cellular processes including protein transport, cell division, and neurotrophic support, but also modulates voltage-gated ion channels activity and the affinity of several neurotransmitters for their receptors (Gardiner and Marc 2010). Here we aim to review novel mechanisms possibly implicated in epilepsy and hyperexcitability states involving alterations to the neuronal cytoskeleton, with particular attention to neurodevelopmental trajectories. In particular, we begin with an overview of how microtubule machinery is organized in neurons. Then, we carry out the description of microtubules dysfunction and its putative influence on neurodegeneration and hyperexcitability states, focusing on the importance of this eventual impairment during neurodevelopment. Overall, we present new intriguing mechanisms, which, if confirmed, could add insights to the topic and represent a target for new therapeutic strategies.

## Basics of Microtubule Organization in Central Nervous System

The neuronal cytoskeleton constitutes a transport track comprising microtubules (MT), actin filaments (AF), intermediate filaments (IF), and associated proteins (AP) (Dent et al. 2010). It serves the intracellular mobilization of molecular cargos and organelles, modulating fundamental operations such as neuronal migration, neuritic growth and synaptic transmission (Conde and Cáceres 2009; Dent et al. 2010; Franker and Hoogenraad 2013; Sánchez-Huertas et al. 2016). Motor proteins driving neuronal transport direct intracellular cargos along either AF or MT (Dent et al. 2010; Franker and Hoogenraad 2013). MT, localized in the cell body and axonal and dendritic compartments, play an important part during neurogenesis and in mature neurons (Conde and Cáceres 2009; Compagnucci et al. 2016). MT serve for long-range transport into distal axons and dendrites, providing lines for kinesin and dynein motor proteins, respectively, to the microtubule plus-end or minus-end (Vale 2003; Hirokawa et al. 2009; Hoogenraad and Bradke 2009). Specifically, current live-cell evidence unveiled the presence of MT not only in the dendritic shaft but also in the spines, serving as specialized dendritic micro-domains fundamental as a wired switch for excitatory inputs (Sheng and Hoogenraad 2007; Jaworski et al. 2009). MT dynamically structure the cells, thanks to the polymerization of α-β-tubulin heterodimers that can be assembled in both parallel and anti-parallel configurations, thus determining MT intrinsic polarity (Sánchez-Huertas et al. 2016). In detail, MT position their plus ends away from the soma in axons, providing a unipolar organization that regulates anterograde and retrograde transport (Hirokawa et al. 2009; Hoogenraad and Bradke 2009). Whereas, in dendrites they arrange into bundles of mixed polarity in which plus ends are predominantly towards the soma, managing bidirectional cargo transport (Poulain and Sobel 2010). This finely regulated MT scaffolding underpins the typical morphology of specific neurons, their compartmentalization and paves the way for neuronal trafficking rules (Kapitein and Hoogenraad 2011; Rolls 2011).

Templates of tubulin ring complexes usually cap only the minus ends of MT; therefore, they tend to elongate only at their plus ends. This establishes the bases of the typical microtubule dynamic instability, ranging from the growing and shrinking of MT plus ends (Kapitein and Hoogenraad 2011). The precise MT orientation is essential for microtubule-based transport since the intrinsic polarity of MT is regulated by neuronal components driving transport either to the plus or minus ends (Kapitein and Hoogenraad 2011). Indeed, MT network is dependent on the strict control of microtubule dynamicity by numerous microtubule-associated proteins (MAP) that are responsible for MT assembly (depolymerization, polymerization, or fragmentation), transport, and stabilization (Poulain and Sobel 2010). In most non-neuronal cells, tubulin dimers and microtubule polymers require a rapid dynamic balance (Fanara et al. 2010). Whereas, in neurons, axonal and dendritic MT proved to be less dynamic in their rapid turnover due to the interaction with a specific subclass of microtubule-associated proteins that maintain the integrity of the microtubule-based axonal transport (Fanara et al. 1999, 2007). Stabilization of hyperdynamic MT is found to be related to neuroprotection in motor neuron degeneration (Fanara et al. 2007). Equally important to sustain neuronal health is tau, a MAP involved in axonal transport and microtubule stabilization. Tau oscillates between MT-bound and MT-unbound state for microtubules normal functioning, in a balance controlled by partially phosphorylated state of tau itself, by kinases and phosphatase enzymes (Morris et al. 2011; Lee and Leugers 2012).

All considered MT appear of core importance for the bioelectric features of neurons since they act as wires that biologically transmit and amplify electric signals via the flow of condensed ion clouds (Craddock et al. 2010). Thus, MT dynamics may have a specific function for neural development. Even if the role of microtubule dynamics on neuroexcitability and synaptic communication has been already suggested (Whatley and Harris 1996; Gardiner and Marc 2010), only recently investigations have pointed at elucidating whether this may directly contribute to epileptic phenomena (Carletti et al. 2016b). However, changes upon MT dynamics in the epileptic foci undoubtedly deserve further examination.

## Impairment of Microtubules in Neurodegeneration and Influence on Epilepsy

The causal relationship between MT transport impairments and neural degeneration is still to uncover (Franker and Hoogenraad 2013; Millecamps and Julien 2013). It seems that damages to the intracellular transport can lead to vesicle-trafficking impairments, alterations of specific cargo interactions and defects in retrograde survival signals, with the ultimate result of neuronal death and loss of brain function (Saxena and Caroni 2007; Perlson et al. 2010). Indeed, impairments of neuronal transport pathways have been reconducted to axonal pathologies underlying Huntington’s, Parkinson’s and Alzheimer’s diseases (Millecamps and Julien 2013), shedding new light on the dangerous linkages between aging and neuronal communication (Akhmedov et al. 2013). Identifying gene mutations upon molecules involved in neuronal transport pathways has brought about the idea that defective transport can directly trigger neurological diseases. For instance, detrimental mutations in α- or β-tubulin and microtubule-based motor proteins were uncovered and implicated in central and peripheral neurological diseases (Tischfield et al. 2010; Millecamps and Julien 2013; Yuen et al. 2019).

**Fig. 1:**
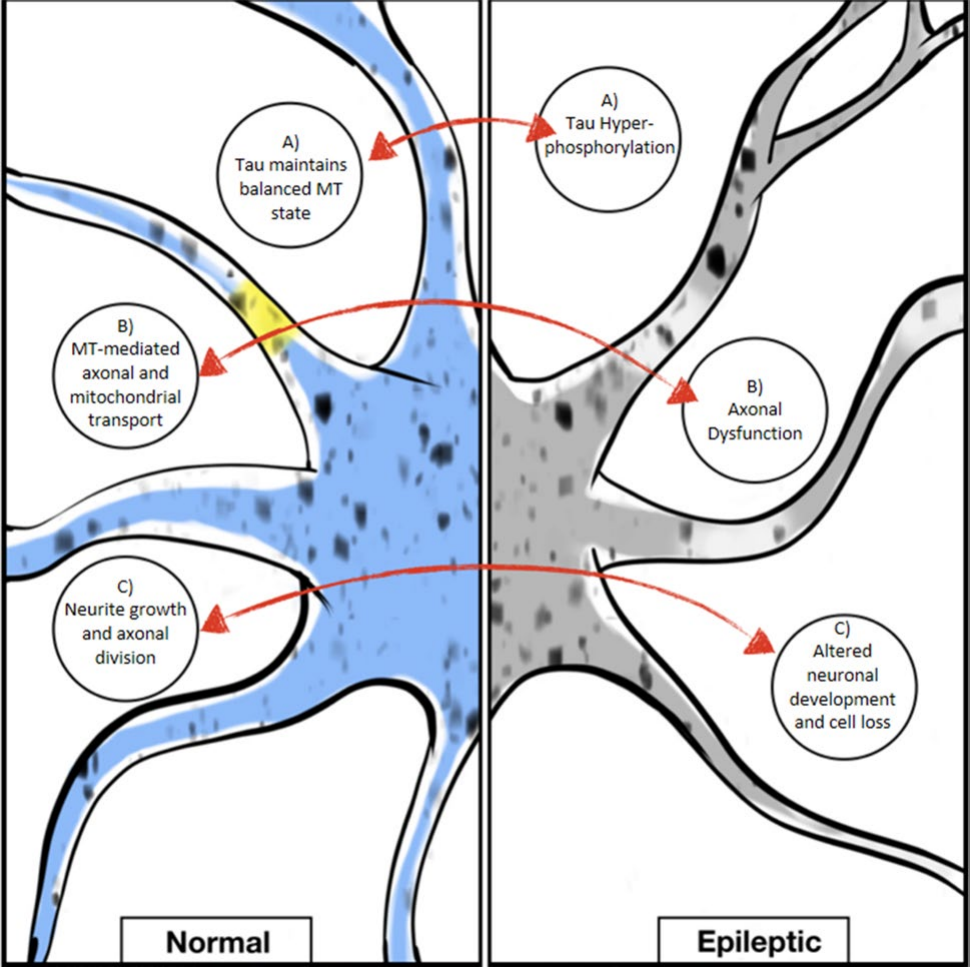
Schematic representation of the main targets implicated in microtubule-mediated actions in normal versus epileptic neurons. (A) In epileptic neurons, it was found tau in hyper-phosphorylated state which is linked to aberrant network organization, whereas in normal neurons tau contributes to balanced microtubule (MT) state and proper functioning. (B) In epileptic neurons, axonal dysfunctions are found, whereas in normal neurons microtubule-mediated axonal and mitochondrial transport are maintained. (C) In epileptic states, microtubules are implicated in altered neuronal development, eventually leading to neuronal loss, whereas in normal neurons MT contribute to proper neurite growth and axonal division

Recently, investigations have been conducted on epileptic disorders and eventual implications of the impairment of neuronal cytoskeleton were explored applying microtubule-active agents that targeted key mechanisms relating microtubule dysfunction and epileptogenesis (Fig. 1). In a pathological environment, alterations to tau and the so-called “tauopathies” play a major role in neurodegeneration (Lee and Leugers 2012; Gunhild et al. 2015; Tai et al. 2020). Indeed, the neuronal balance in certain cases shifts to hyper-phosphorylation of tau that does not bind anymore to MT, thus leading to cytoskeletal destabilization, axonal dysfunctions and ultimately, if cell viability is irreversibly blocked, to neuronal apoptosis (Lee and Leugers 2012; Kondo et al. 2015). Tau hyper-phosphorylation is a pathological feature observed in brain samples from epileptic patients (Sen et al. 2007; Puvenna et al. 2016; Liu et al. 2017) and its pharmacological manipulation provided anti-seizure effects in several chronic acquired models of epilepsy (Tian et al. 2010; Jones et al. 2012; Garg et al. 2018). For instance, Liu et al. (Liu et al. 2016) have recently proved that treatment with an inhibitor of tau phosphorylation protects from seizure induction and paroxysmal phenomena in acute and chronic models of epilepsy, as per other authors (Holth et al. 2013; Devos et al. 2013). The correlation of hyper-phosphorylated tau with epilepsy was also specifically located in the temporal lobe and associated with cognitive decline in patients behaviorally assessed before temporal lobe resection (Tai et al. 2016). Molecularly, tau hyper-phosphorylation is considered to trigger aberrant network re-organization such as mossy fibers sprouting, putatively mediated by NR2B subunit of NMDA glutamate receptors, which is deemed as a fundamental marker for epilepsy development (Tian et al. 2010; Decker et al. 2016). Furthermore, the network hyperexcitability associated with tau modification can implicate, among the proposed mechanisms, the enhancement of presynaptic glutamate release (Vossel et al. 2016, 2017). In line with this, seizure-induced neuronal loss and axonal damage may lead to the development of aberrant connections between limbic structures and eventually result in the reorganization of the limbic network (Spencer 2002). Lastly, tau deletion has been associated with reduced severity of tumor-related epilepsy, together with a functional dysregulation of glutamate homeostasis (Hatcher et al. 2020).

Microtubule cytoskeleton seems strongly implicated in seizure-dependent network remodeling since the expression of α-tubulin rises, together with several MAPs and hyper-phosphorylated tau, within the sprouting of mossy fibers (Pollard et al. 1994; Gardiner and Marc 2010; Tang et al. 2014). In detail, hippocampal kindling in the rat was associated with aberrant synaptic reconstruction, due to the increase in the dentate gyrus of MAP-2, a structural protein determining the amount of MT in dendrites (Tang et al. 2014). As well, augmentation of α1-tubulin mRNA levels was found in the kindled hippocampus of rodents, together with sprouting and reorganization of neural networks (Sato and Abe 2001). In addition, the up-regulation of genes associated with tubulin has been found in the human brain from patients with drug-resistant focal epilepsy (Xi et al. 2011; Machado et al. 2019). A relevant study outlined that several kinesin-knockout mice are characterized by epileptic phenotype evidenced by electroencephalogram abnormalities. The authors explained this considering the impairment of GABA A receptor-mediated synaptic transmission (Nakajima et al. 2012). Other studies showed that augmented gene expression of α-tubulin and an altered microtubule formation in kainic acid model of epilepsy contributed to aberrant neurogenesis in the hippocampus (Represa et al. 1993; Hendriksen et al. 2001). Nonetheless, the exact implication of aberrant tubulin has not been clarified yet, since a down-regulation of both α- and β-tubulin proteins was found in mesial TLE (Yang et al. 2006), due to the alteration of tubulin acetylation by the deacetylase Sirtuin 2. Among the few studies on the epileptic brain, it was also observed the unbalance of actin cytoskeleton assembly and disassembly following status epilepticus. In acute and chronic chemical models of epilepsy the acute depolymerization and long-term remodeling of F-actin were found, thus potentially contributing to the process of epileptogenesis (Zeng et al. 2007; Zhang et al. 2014). Recently, the use of dexamethasone, known to control actin polymerization, reduced the damage to actin filaments in pilocarpine-induced temporal lobe epilepsy (Yang et al. 2019). It is worth noting that infantile epileptic syndromes characterized by spasm (West syndrome) have been empirically treated with steroids since the ‘60 s. (Chutorian et al. 1968). More recently, steroids have been used to treat status epilepticus in slow-wave sleep (ESES) (Moosa 2019). However, treatment with steroids is not effective in other forms of epilepsy (Mehta et al. 2015). This suggests that they only act on a specific etiologic mechanism rather than on epilepsy per se. On this point, it has been reported a mutation on PHACTR1, a molecule critical for the regulation of protein phosphatase 1 and the actin cytoskeleton, in patients with West syndrome (Hamada et al. 2018).

In the light of these data, it seems that pharmacological manipulation of cytoskeletal and microtubule functioning could be intriguingly relevant to the development and the control of epilepsy, as summarized in Table 1. Previous study provided information that davunetide, preserving microtubule dynamics, can reduce kainate toxicity (Zemlyak et al. 2009). To this purpose, previous data explored the genesis and maintenance of epileptic activity when perturbing the microtubule assembly process in the hippocampus, in both intact neuronal network and isolated brain slices in vivo and in vitro in the rat (Carletti et al. 2016b). The main novelty of the study was represented by the choice of pharmacological molecules widely administered in cancer therapy as anti-mitotic such as nocodazole and paclitaxel (Jordan and Wilson 2004). This paper showed for the first time that nocodazole protects and modulates electrophysiological paroxysmal activity in rat TLE models, by reducing the onset and duration of epileptic discharge in vivo and also of hippocampal bursting activity in vitro (Carletti et al. 2016b). On the other hand, it was also described a case of non-convulsive status epilepticus induced by chemotherapeutic application of paclitaxel and discovered by electroencephalographic examination of an oncologic patient (Illán-Gala et al. 2015). The pharmacokinetic interaction of AEDs with chemotherapeutic drugs has been already outlined and ascribed to the susceptibility of cytochromes (CYP) enzymes to drug inducers or inhibitor, but complex effects of microtubule agents on seizures could hint a more specific interaction (Bénit and Vecht 2016). Other studies investigated MT-active agents in neurodegenerative diseases. For instance, it was found a protective role for paclitaxel in Alzheimer’s diseases. Indeed, this MT-stabilizing agent, as well as other taxanes with comparable potency in promoting tubulin assembly, protects against amyloid-β (Aβ) toxicity and enhances neuronal survival, at low doses (Michaelis et al. 2005). Also in Huntington’s neurodegeneration, paclitaxel treatment acted on an early step of MT destabilization remarkably boosting survival of primary striatal neurons expressing a toxic mutant protein (Trushina et al. 2003). It could be counterintuitive that cytotoxic drugs support neuronal survival as in (Michaelis et al. 2005; Carletti et al. 2016b), in fact these drugs can interfere with processes initiated by excessive stabilization of the cytoskeleton and loss of dynamic instability. Furthermore, blocking early MT depolymerizing signals associated with several insults, could be a way to trigger cascades that enhance cell survival, acting as a supervising system for cell destiny. This innovative viewpoint on epileptogenesis has been supported by a growing number of authors focusing on the reduced axonal and dendritic transport of cargos such as mitochondria that physiologically regulate neuronal excitability (Vossel et al. 2010, 2017; Morris et al. 2011).

**Table 1:** Microtubule-active agents in epileptic states

Research papers	Studies on humans	Studies on animal models	Microtubule-active agents applied	Main effects obtained
Jones et al. (2012)	X	Pentylenetetrazole rat model	Sodium Selenate	Tau Dephosphorilation, neuroprotection
Liu et al. (2016)	X	Pentylenetetrazole rat model	Sodium Selenate	Tau Dephosphorilation, neuroprotection
Devos et al. (2013)	X	Pentylenetetrazole mouse model	Central oligodeoxynucleotides	Reduction of Tau levels, neuroprotection
Holth et al. (2013)	X	In vitro mice epilepsy model	Tau genotyping	Reduction of Tau levels, neuroprotection
Yang et al. (2006)	Yes	X	X	Down-regulation of tubulins associated with epilepsy
Zeng et al. (2007)	X	Kainate mouse model	Calcineurin inhibitor	Stabilization of actin dynamics reduces seizures
Yang et al. (2019)	X	Pilocarpine mouse model	Dexamethasone	Control of actin polymerization reduces seizures
Zemlyak et al. (2009)	X	Kainate mouse model	Davunetide	Protection of microtubule functioning and reduction of kainate neurotoxicity
Carletti et al. (2016b)	X	In vivo and in vitro rat epilepsy models	Nocodazole, Paclitaxel	Modulation of microtubule polymerization and neuroprotection exerted by nocodazole
Illán-Gala et al. (2015)	Yes	X	Paclitaxel	Induction of non-convulsive status epilepticus

### Neurodevelopmental Aspects of Microtubule Dysfunction and Epileptogenesis

The overviewed importance of microtubule-dependent functioning in neuronal hyperexcitability could sink deeper roots early in the neurodevelopment of synapses (as summarized in Table 2). The modulation of key synaptic factors during neurodevelopment can be considered as a protective strategy against epileptogenesis (Aronica et al. 2011; Grabenstatter et al. 2014). Microtubule machinery plays a prominent role in neurodevelopment and is finely regulated by mechanisms like phosphorylation, acetylation, tyrosination, and glutathionylation (Compagnucci et al. 2016). MT helps the developing brain in the navigation and formation of axons (Franker and Hoogenraad 2013; Mutch et al. 2016). Even during the process of mitosis, MT migrate from the centrosome and modulate cell division by attachment to chromosomes. In this context, MAPs are particularly useful for neuronal migration, helping the nucleokinesis, in axonal navigation (Huang et al. 2012; Liu and Dwyer 2014) and mitochondrial biogenesis (Augustyniak et al. 2017). Indeed, the implication of MAP 2 on the efficiency of mitochondria in neuronal differentiation has been examined for the impact on neurodegenerative disorders (Augustyniak et al. 2017). Additionally, neuritogenesis, axonogenesis, and dendritogenesis are essential processes relying on the specific dynamics of the microtubule cytoskeleton, for instance microtubules polarized ends are differently arranged from immature neuritis to mature dendrites (Poulain and Sobel 2010). Some specific tubulin binding proteins, for instance belonging to stathmin family, were found to regulate axonal branching without affecting growth cone size (Poulain and Sobel 2007). All considered, microtubule transport not only supports the restructuring of membranes, organelles and macromolecules but also contributes to the elaboration of dendritic trees (Sharp et al. 1997).

**Table 2:** Role of microtubule dysfunction on neurodevelopment

Research papers	Target studied	Main effects evidenced
Franker and Hoogenraad (2013), Huang et al. (2012), Liu and Dwyer (2014)	MAP	Axon formation and navigation
Augustyniak et al. (2017)	MAP 2	Mitochondrial biogenesis
Poulain and Sobel (2007)	Stathmin 2	Neuritogenesis, axonogenesis and dendritogenesis
de Nijs et al. (2006)	EFHC1	Altered cell division and juvenile myoclonic epilepsy
Xia et al. (2003), Nakajima et al. (2012)	KIF5A	Alteration on GABA A functioning and predisposition to seizures

These orchestrated events, when impaired, lead to malformations of a functional neuronal network, usually evidenced by cognitive deficits and neurodevelopmental disorders such as microencephaly, lissencephaly, polymicrogyria, and infantile epileptic syndromes (Cushion et al. 2014; Compagnucci et al. 2016). Among the numerous proteins that were implicated in these processes, mutations of EFHC1 protein have been related to juvenile myoclonic epilepsy (de Nijs et al. 2006). This protein accumulating at the centrosome during interphase, is fundamental for cell division. Also kinesin proteins undergo neurodevelopmental modifications leading to hyperexcitability such as the postnatal loss of kinesin gene KIF5A predisposing to seizures (Xia et al. 2003). When KIF5A is deficient in neurons, EEG recordings evidenced epileptic seizures probably due to impaired GABA A trafficking (Nakajima et al. 2012). Importantly related to neurodevelopmental alterations of microtubules is a brain-derived neurotrophic factor (BDNF), trafficked one way and back along microtubules between the cell periphery and the nucleus (Butowt and von Bartheld 2007). Some authors assert that BDNF favors neuronal survival and growth in epileptic rats (Yu et al. 2019), whereas others agree that excessive expression of BDNF is involved in the pathogenesis of hippocampal hyperexcitability and epilepsy (Gardiner and Marc 2010). Overall, the genetic implication of neurotrophic factors and neurodevelopmental impairments could represent a target for studies on long-lasting modifications of synaptic plasticity and the related morphological changes of synapses. This could bring awareness on the importance of monitoring and preventing microtubules dysfunction eventually triggering pathophysiological alterations of neuronal activity.

## Novel Effects of Microtubules on Neuronal Excitability: Role of Mitochondria and Cannabinoids

A deeper insight on minute effects of microtubules functioning within neuronal machinery can be gained considering the most recent advances on the field, i.e. the role of mitochondrial transport in microtubule-mediated control of neuronal activity. The proper, unceasing mitochondria trafficking within the neuron is indeed essential to fulfill energy requirements of neurons for instance to efficiently perform neurosignalling or to re-establish ion gradients (Ogawa et al. 2016). Trafficking of neuronal mitochondria in both axons and dendrites is functional to the proper activity such as basic energy supply, but also to their degradation when damaged or aged. In this processes, many MT motors and adaptor proteins support the distribution to precise cellular compartments, the flux and movement of neuronal mitochondria (Melko and Abdu 2018). In particular, several microtubule cytoskeleton-associated proteins are involved in the axonal mitochondrial transport such as TRAK1 and DISC1, whose dysfunction has been studied for putative impact on neuronal impairment and possibly on pathologies of the CNS (Manji et al. 2012). It seems that mitochondria are transported in the anterograde direction by kinesins (Pereira et al. 1997), while dynein might be their retrograde motor (Habermann et al. 2001). However, the exact way microtubule motors are concertedly regulated to guarantee mitochondrial transport is still a matter of debate. It has been suggested that axonal transport of mitochondria is fundamental to maintain a uniform distribution of them throughout the neuron, to the ultimate purpose of proper neuronal efficiency. Specifically, functional mitochondria with high potential are transported out into the axon, while the old or damaged ones with low potential are transported back to the cell body (Miller and Sheetz 2004). For instance, an eventual impairment in MT-mediated transport could be targeted as a precursor of a progressive accumulation of damaged mitochondria, which is typical of neurological conditions like Alzheimer’s disease (AD, Ye et al. 2015).

Considering that neuronal cells are extremely sensitive to even mild changes in mitochondrial dynamics, intriguing researches have recently investigated the role of cannabinoid receptors on mitochondrial activity in the brain (Hebert-Chatelain et al. 2014). Indeed, the cannabinoid system represents a widely known endogenous repertoire that is implicated in a plethora of pathophysiological functions, such as learning and memory, neuronal excitability and neuroadaptation (Marsicano et al. 2003; Ligresti et al. 2016). Within the context of neuronal excitability, researches support the neuroprotection exerted by cannabinoids on bioelectric balance in neurons (Carletti et al. 2017; Gambino et al. 2020). This protection could be also exerted on the maintenance of cytoskeletal dynamics, considering that activation of cannabinoid receptors type 1 (CBr1) has been associated with cytoskeletal reorganization to the purpose of stabilizing axonal morphology (Tortoriello et al. 2014).

The discovery of specific localization of CBr1 within CA1 hippocampal mitochondrial membranes sets out a novel path in the experimental field of neuroexcitability (Hebert-Chatelain et al. 2014; Busquets-Garcia et al. 2015). Indeed, Hebert-Chatelain et al. (Hebert-Chatelain et al. 2016) demonstrated that mitochondrial CBr1 triggers a cascade of events decreasing brain mitochondrial activity, necessary for the acute effects of cannabinoids on mitochondrial mobility, synaptic depression and eventually memory impairments. For instance, in hyperexcited states, cultured hippocampal neurons treated with cannabinoid agonist showed higher Ca^2 +^ uptake by mitochondria (Ryan et al. 2009). Cannabinoid-mediated mitochondrial processes could also be influenced by a Ca-dependent endogenous mediator, nitric oxide (NO; Tedesco et al. 2010; Lipina et al. 2014).

The suggested alteration of intracellular activity is of striking importance when considering eventual long-term impact on the pathogenesis of neurological disorders. Considering all this, it could be conceivable an influence of CBr1, on the transport and activity of mitochondrial influencing excitability of the cells, putatively involving elements associated with neuronal transport such as microtubules. Targeted studies will be needed to explore this fascinating hypothesis here formulated to explore the contribution of cannabinoids and microtubule motors on mitochondrial activity to investigate the influence on neuronal excitability.

## Conclusive Remarks

The present review provides a snapshot on cytoskeletal components implicated in the pathogenesis of neurological disorders and, above all, of epileptic conditions. Increasing attention has been dedicated to the microtubule cytoskeleton in the last years for its compelling coordination of complex elements of neuronal transport, ultimately regulating synaptic activity.

Here we propose a neurobiological perspective of the possible targets orchestrated by microtubules functioning with minute molecular details comprising, among the others, mitochondrial transport and cannabinoid-mediated effects. The neurodevelopmental etiology of microtubule dysfunction was examined to explore the early stages of neuronal impairment and to individuate eventual chances of therapeutic intervention. Nonetheless, the ultimate goal was to evaluate the translational application of manipulating microtubule dynamics in the alteration of neuronal excitability and the related, not-well pharmacologically controlled pathologies such as epilepsy.
